# 

**Published:** 1862-11

**Authors:** 


### CONTENTS.

ORIGINAL ESSAYS AND COMMUNICATIONS.

Influence of the times upon the Dental Profession. By J. Taft........485
Success in the Dental Profession By C. C. Dills........487
Future of Dentistry. By J. Taft........492

CORRESPONDENCE.

History of Dentistry on the Pacific Coast........495

SELECTIONS.

Dentition and its Derangements........498
A New Method of Regular and Constant Coaertation of parts in Operations for Harelip...511
Neuralgia of the Teeth........513
Some of the effects of Tobacco........522

EDITORIAL.

Medical Specialities........525
A Peculiar Case—No. 5........527
Woods Plastic Metallic Fillings........528
Dental Association........529
American Journal of Ophthalmology........530
A Case—Uncontrollable—No. 6........531
Block Teeth........532
Causes and Cure of Diseases of the Feet, with practical suggestions as to their clothing..532

